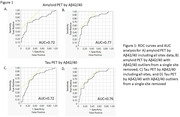# Alzheimer’s Disease Plasma Biomarker Results from across 14 Alzheimer’s Disease Research Centers

**DOI:** 10.1002/alz.092540

**Published:** 2025-01-09

**Authors:** Kristen A. Russ, Sanjay Asthana, Sterling C. Johnson, Rachael E Wilson, Suzanne Craft, Thomas C. Register, Samuel N. Lockhart, Angus C Nairn, Stephen M Strittmatter, Christopher H. van Dyck, Tatiana M. Foroud, Jeffrey L. Dage

**Affiliations:** ^1^ Indiana Alzheimer's Disease Research Center, Indianapolis, IN USA; ^2^ Indiana University School of Medicine, Indianapolis, IN USA; ^3^ Wisconsin Alzheimer's Disease Research Center, School of Medicine and Public Health, University of Wisconsin‐Madison, Madison, WI USA; ^4^ Wisconsin Alzheimer's Disease Research Center, Madison, WI USA; ^5^ Wisconsin Alzheimer's Disease Research Center, University of Wisconsin School of Medicine and Public Health, Madison, WI USA; ^6^ Wake Forest Alzheimer's Disease Research Center, Winston‐Salem, NC USA; ^7^ Wake Forest University School of Medicine, Winston‐Salem, NC USA; ^8^ Yale School of Medicine, New Haven, CT USA; ^9^ Alzheimer's Disease Research Unit, Yale School of Medicine, New Haven, CT USA; ^10^ Stark Neurosciences Research Institute, Indiana University School of Medicine, Indianapolis, IN USA; ^11^ Stark Neurosciences Research Institute, Indianapolis, IN USA

## Abstract

**Background:**

The Alzheimer’s Disease Center Fluid Biomarker (ADCFB) Initiative samples are analyzed centrally at NCRAD for AD plasma biomarkers. When combining NACC accessible data from across centers, biofluid biomarker data must be evaluated carefully. This will become more critical with the implementation of disease modifying therapies.

**Methods:**

Beta amyloid 1‐42 (Aβ42) and beta amyloid 1‐40 (Aβ40) were analyzed utilizing the Neurology 4‐Plex E kits on a Quanterix Simoa HD‐X. All assays were performed according to manufacturer’s instructions. NACC data from participants 65 or older was combined with biomarker results into one data set. Samples with PET results from the same visit as the blood collection were utilized for this analysis (n=114).

**Results:**

Data for amyloid and tau PET was used along with Aβ42/40 ratios to assess the area under the curve (AUC) for this data set (Figure 1). Amyloid PET and Tau PET by Aβ42/40 ROC analysis including age and APOE4 carrier status showed lower than expected AUCs (both 0.72). A subset of data (n=90) was analyzed using participants that were not on any FDA‐approved drugs for AD. This had no effect on AUCs for amyloid or tau PET by Aβ42/40 ratios. Distribution of Aβ42/40 ratios across sites showed a single site had a subset of very high Aβ42/40 ratios (n=8) in comparison to other sites. After removal of the Aβ42/40 outliers from the specific site from the data set, diagnostic accuracies of Aβ42/40 for both Amyloid PET (AUC=0.77) and Tau PET (AUC=0.76) were increased. More investigation into the exact cause of the outliers is necessary, but Aβ42/40 elevations independent from other biomarkers have been seen in clinical trials of Solanezumab and some other Aβ targeting antibodies.

**Conclusion:**

To avoid errors in data analysis when using shared data, it is important to track clinical trial co‐enrollment and drug type within ADCs at NACC. As FDA‐approved treatments become available or co‐enrollment of AD drug trials at centers occurs, it is critical to carefully track participant variables and review biofluid biomarker data when it is being combined across centers or studies.